# Prevalence and associated factors to Post-Traumatic Stress Disorder (PTSD) and Social Anxiety Disorder (SAD) among health workers in the emergency room

**DOI:** 10.1192/j.eurpsy.2023.1425

**Published:** 2023-07-19

**Authors:** B. Amamou, I. Betbout, A. Ben Haouala, M. Ben Mbarek, I. Merchaoui, R. Jebali, F. Zaafrane, L. Gaha

**Affiliations:** Psyciatry, faculty of medicine of Monastir, university of monastir, Monastir, Tunisia

## Abstract

**Introduction:**

post-traumatic stress disorder (PTSD) is a mental health illness that can develop after being exposed to one or more traumatic events. This is a serious, long-term emotional response to extreme psychological trauma. As for public health emergencies, it demands large-scale coordination among many staff, and participants, especially medical workers, are exposed to high levels of stress, which can easily lead to psychiatric illnesses such as social anxiety disorder (SAD). Posttraumatic stress disorder (PTSD) and social anxiety disorder (SAD) demonstrate a high degree of comorbidity, yet little is known regarding the nature of this relationship.

**Objectives:**

The aim of this study is to investigate the prevalence of PTSD and SAD among health workers in the emergency ward and study the relationship between PTSD and SAD and the associated factors to both disorders among health workers in the emergency ward to suggest some solutions to reduce their effects.

**Methods:**

This is a quantitative descriptive cross-sectional study conducted among medical and paramedical health professionals in the emergency rooms of the university hospital of Sahloul and Hached and the regional hospital of Msaken in Tunisia. The data was collected by a questionnaire that included demographic questions and Yes/No questions, as well as several scales to assess the degree of social nxiety (Liebowitz Social Anxiety Scale) and posttraumatic stress disorder (Post-traumatic stress disorder checklist for DSM-5 (PCL-5)).

**Results:**

In our study, 81 healthcare workers completed the survey. Of the total responding participants 67.9% were females. We noticed that the example was young (58%), also 59.3% had <1 year of experience. The population was slightly predominated by paramedical staff (56.8%), it also had a low married percentage of 38.3. Among the participants 17.3% smoke tobacco, 12.3% drink alcohol, and 3.7% are under cannabis use. We found that 7.4% of the participants had a psychiatric illness.

In our study, 38% scored positive for PTSD and for SAD 13.58% had marked social anxiety, 12.35% had severe social anxiety and 3.7% had very severe social anxiety, this is associated rather with the female gender, the younger (age range 20-30 years) and the paramedical staff.

PTSD and SAD are more pronounced among those with the fewest years of experience.

We obtained a positive Pearson Correlation between PTSD and SAD (r=0.513).

**Conclusions:**

Posttraumatic stress disorder (PTSD) and social anxiety disorder (SAD) demonstrate a high degree of comorbidity, especially in the healthcare field. Overall, researchers reveal that the link between PTSD and SAD is complicated, owing to a variety of factors such as a person’s genes, trauma history, and psychological vulnerabilities so large-scale epidemiological investigations are required.

**Disclosure of Interest:**

None Declared

